# A Case of Serious Effusion in the Theca of the Medulla Spinalis

**Published:** 1847-12

**Authors:** 


*A case of Serious Effusion in the Theca of the Medulla Spinalis.* By H. T. CHILD, M. D.—I was requested on the morning of the 11th inst., to see R. D. S., male, aged twenty-six. I found him sitting up, and obtained the following history of his case:—He had been exposed to a severe rain, two days previously; and on the afternoon of the 10th he was attacked with paralysis of the lower extremities, so suddenly that he fell over while engaged at business. When I saw him at 9 A. M., the paralysis had extended so as to partially interfere with the motions of the upper extremities; sensation appeared natural; his pulse was about 80; there was no apparent tenderness on pressure along the spine; he had passed his urine freely within an hour; his speech was somewhat affected. I directed a cathartic of extract of Colocynth combined with powdered Cantharides.

At 3 P. M., my friend Dr. C. H. Bibighaus saw him with me, and we found that, soon after I left, he had lost the power of deglutition; his pulse was now 88, and rather oppressed; we directed free cupping along the spine and a blister to the nape of the neck; in the evening the pulse was 100; he had swallowed nothing, but was profusely salivated and had been all day, the cause of which we were unable to trace. I emptied the bladder by means of the catheter, and directed an enema of infusion of senna and tinct. of aloes.

On the morning of the 12th, Dr. Noble saw him in company with Dr. B., and myself; his pulse was 90; he had had his bowels evacuated by the enema and had passed his urine ; his mind was somewhat affected; sensation natural; constant restlessness and desire to be moved. We directed sixteen ounces of blood to be taken, by cups to the spine, and a repetition of the enema. At 1 o'clock, P. M., his pulse was 120; skin hot and dry. I passed a tube into his stomach, and injected a gill of milk and water; he said he felt refreshed; from this time he sank rapidly; his breathing became hurried and stertorous, and at 7 P. M., he died, about 50 hours after the first appearance of disease.

A post mortem examination was made thirty-six hours after death, by Dr. Bibighaus and myself—Dr. Noble being absent from the city. We opened the spinal column in the lumbar region, and found it filled with transparent serum; the theca was somewhat softened, and there was atrophy of the medulla spinalis. We were not able to examine the condition of the brain and other viscera.—*Medical Examiner*

